# The Effect of Zinc Oxide on DLP Hybrid Composite Manufacturability and Mechanical-Chemical Resistance

**DOI:** 10.3390/polym15244679

**Published:** 2023-12-12

**Authors:** Janis Baronins, Maksim Antonov, Vitalijs Abramovskis, Aija Rautmane, Vjaceslavs Lapkovskis, Ivans Bockovs, Saurav Goel, Vijay Kumar Thakur, Andrei Shishkin

**Affiliations:** 1 Laboratory of Ecological Solutions and Sustainable Development of Materials, Faculty of Materials Science and Applied Chemistry, Institute of General Chemical Engineering, Riga Technical University, Pulka 3, K-3, LV-1007 Riga, Latvia; vitalijs.abramovskis@edu.rtu.lv (V.A.); aija.rautmane@rtu.lv (A.R.); vjaceslavs.lapkovskis@rtu.lv (V.L.); andrejs.siskins@rtu.lv (A.S.); 2 Latvian Maritime Academy of Riga Technical University, Riga Technical University, Flotes Str. 12 K-1, LV-1016 Riga, Latvia; 3 Department of Mechanical and Industrial Engineering, Tallinn University of Technology, Ehitajate Tee 5, 19086 Tallinn, Estonia; maksim.antonov@taltech.ee; 4 Faculty of Materials Science and Applied Chemistry, Institute of Polymer Materials, Riga Technical University, 3/7 Paula Valdena Street, LV-1048 Riga, Latvia; ivans.bockovs@rtu.lv; 5 School of Engineering, London South Bank University, London SE1 0AA, UK; goels@lsbu.ac.uk; 6 Department of Mechanical Engineering, University of Petroleum and Energy Studies, Dehradun 248007, India; 7 Biorefining and Advanced Materials Research Center, Scotland’s Rural College (SRUC), Kings Buildings, West Mains Road, Edinburgh EH9 3JG, UK; vijay.thakur@sruc.ac.uk

**Keywords:** DLP, additive manufacturing, ZnO, photocured resin, tensile test, corrosion, acidic environment, alkaline environment

## Abstract

The widespread use of epoxy resin (ER) in industry, owing to its excellent properties, aligns with the global shift toward greener resources and energy-efficient solutions, where utilizing metal oxides in 3D printed polymer parts can offer extended functionalities across various industries. ZnO concentrations in polyurethane acrylate composites impacted adhesion and thickness of DLP samples, with 1 wt.% achieving a thickness of 3.99 ± 0.16 mm, closest to the target thickness of 4 mm, while 0.5 wt.% ZnO samples exhibited the lowest deviation in average thickness (±0.03 mm). Tensile stress in digital light processed (DLP) composites with ZnO remained consistent, ranging from 23.29 MPa (1 wt.%) to 25.93 MPa (0.5 wt.%), with an increase in ZnO concentration causing a reduction in tensile stress to 24.04 MPa and a decrease in the elastic modulus to 2001 MPa at 2 wt.% ZnO. The produced DLP samples, with their good corrosion resistance in alkaline environments, are well-suited for applications as protective coatings on tank walls. Customized DLP techniques can enable their effective use as structural or functional elements, such as in Portland cement concrete walls, floors and ceilings for enhanced durability and performance.

## 1. Introduction

In various production processes, epoxy resin (ER) is widely utilized by industries due to its superior adhesion, ability to eliminate air bubbles, and excellent electrical and thermal insulating properties [1,2,3]. The rigid molecular structure of the ER matrix provides high thermal stability and mechanical strength [4]. As global initiatives focus on sustainable practices, the European Union Green Deal and worldwide objectives for zero-emission industries emphasize the importance of using manufacturing waste and recycled materials in composites [5].

Additive manufacturing, particularly 3D printing, is a versatile process that can produce a wide range of materials. These include metal oxides such as ZnO, SiO_2_, and TiO_2_, which can be combined with polymers to create new and innovative products [6,7,8]. Various techniques such as intense pulsed light (IPL), digital light processing (DLP), fused deposition modeling (FDM), fused filament fabrication (FFF), and stereolithography (SLA) can be used to manufacture these products. It’s worth noting that while FDM and FFF share a common additive manufacturing approach, distinctions arise with FDM being a trademarked term by Stratasys, and FFF being a more generic, open-source concept that enables wider material and equipment compatibility [9]. Table 1 summarizes key data for composite materials used in 3D printing, showcasing the potential of metal oxides in different applications.

The combination of ER monomers with prepolymers and photosensitive additives enables rapid liquid photocuring into a complex shaped solid product using techniques such as SLA and DLP [10,11]. Ultraviolet (UV) cured coatings and printable materials, known for their environmental safety in adapted working conditions and lower energy consumption, as compared to thermally cured ER analogs, have gained attention [12,13]. UV-curable coatings find applications in shipbuilding, providing corrosion protection and mechanical durability properly grinded surface [14]. However, challenges exist, such as the limitations of UV-cured thin films in barrier (e.g., gas, and solvents) properties and mechanical strength [15,16].

Polyurethane acrylate (PUA) oligomers, a petrochemical product, play a significant role in UV-cured surfaces [17,18]. Relatively soft UV-cured ER surfaces exhibit low scratch resistance [19,20]. One of the solutions is to increase the polymer matrix hardness. The incorporation of harder additives (e.g., ZnO with typical Vickers hardness from 2 to 4 GPa) [21] into PUA (with hardness from 0.12 to 0.23 GPa) [22] matrix is the simplest approach. It should be noted that Vickers hardness tests are commonly used for ceramics to assess their resistance to indentation, while Shore hardness tests are preferred for polymers to measure their flexibility and elasticity. Comparing the hardness of polymers and ceramics is complicated due to their inherent differences in material properties; polymers are typically more compliant and deformable, while ceramics are significantly more rigid and brittle, making direct comparisons challenging as hardness values are influenced by the materials’ distinct responses to applied stress and deformation.

Despite the historically performed tuning of polyurethane mechanical properties with different nanoparticles, such as nano clay [23], carbon nanotubes [24], metal oxide, hydroxyapatite, and graphene (including its derivatives) [25,26], the market offers limited number of solid fillers adopted for SLA and DLP needs. Industry commonly uses rigid inorganic fillers such as kaolin and silica, but the challenge lies in managing surface roughness at high concentrations [27]. Epoxy composites’ heterogeneity influences physio-mechanical properties [28], and the use of ceramic micro and nanoparticles as fillers aids in reducing shrinkage and warping errors in SLA and DLP products [29,30].

DLP polymers with inorganic fillers, particularly zinc oxide (ZnO), have attracted attention for their improved thermal stability [31], mechanical strength, reduced vapor permeability [32], and enhanced optical and electrical properties [33]. ZnO, classified as an n-type metal oxide [34], exhibits excellent radiation resistance, electrical and optical properties optical (e.g., absorption at local UV spectrum ranges from around 350 to 362 nm) [35], and finds applications in various industries. Industrial production of gas [36] sensors, humidity sensors [37], catalysts [38], and antibacterial materials [39] are some of typical ZnO application examples.

Dispersion of ZnO in PUA oligomers require high energy. Industry and researchers mostly apply in-situ polymerization [40] or blending [41] processes for manufacturing polyurethane-ZnO composites. Manufacturers currently produce liquid PUA oligomers with blended rigid particles. Several researchers have reported the ZnO mechanical stirring and ultrasonic mixing mostly at room temperature. Different approaches have resulted in different required mixing durations and stability of homogeneous dispersions [42,43,44,45]. Unfortunately, the poor compatibility and interfacial interaction between the ZnO particles and liquid PUA oligomers promotes the aggregation and sedimentation of ZnO particles [46]. Conventionally produced PUA exhibits similar corrosion rates in aqueous acidic (5% HCl) and alkaline (5% NaOH) solutions [47]. However, much less corrosion research data is available on additive manufactured PUA products.

**Table 1:** Comprehensive summary of composite materials for 3D printing: metal oxides (ZnO, SiO_2_, and TiO_2_) containing polymer compositions and properties of products.

No.	Filling Substance	Particle Size	Loading	Additive Manufacturing Method	Properties/Applications	Ref.
1	ZnO	0.7 µm	44–52 vol.%	IPL	The compressive strength ranges from 5.08 to 11.09 MPa at temperatures of 900 to 1500 °C.	[48]
2	ZnO	100 nm	38 wt.%	DLP	The compressive strength ranges from 1.26 to 6.82 MPa for materials with a Gyroid structure and Schwartz P structure.	[49]
3	ZnO	<130 nm	10 wt.%	FDM	New devices are continuously emerging for pertinent applications in fields such as environmental science, energy, and catalysis.	[50]
4	ZnO	Highly concentrated ZnO ink	50 vol.%	Robotic deposition equipment	ZnO optoelectronic devices operate at THz frequencies and can be seamlessly integrated with various optical components such as waveguides and resonators.	[51]
5	SiO_2_	100 nm	2 wt.%	IPL	The applicability of inkjet 3D printing in the electronics industry is promising with ink characteristics such as a density of 1.05 g·mL^−1^ and a viscosity of 9.53 mPa·s, enabling precise and controlled deposition of conductive materials for circuit fabrication.	[52]
6	SiO_2_	5–15 nm	0.5–4 wt.%	FFF	Tensile stress ranges from 31 to 35 MPa, with a corresponding tensile modulus of elasticity of 138–148 MPa. Additionally, it has a flexural strength of 40–47 MPa and a flexural modulus of elasticity spanning 786–927 MPa. The impact resistance falls within the range of 3.72–4.01 kJ·m^−2^, and the microhardness measures between 12.44 and 13.34 HV.	[53]
7	SiO_2_	The diameter of the fiber is 6.5 μm. 20 nm powder	10 vol.% (fiber) 3.68–11.76 wt.% powder	Direct ink writing	The composite material exhibits a dielectric constant of 1.2 and a dielectric loss tangent of 1.5 × 10^−2^. Its bending strength ranges from 11.2 ± 1.1 to 14.15 ± 1.3 MPa, while the apparent porosity falls within the range of 24.36% to 24.48%.	[54]
8	TiO_2_	10 nm	0–2.5%	SLA	The material demonstrates a tensile stress between 17 and 25 MPa, an impact resistance of 17.5 to 25 kJ·m^−2^, a hardness of 80 HV, and an elongation at break of 8 to 8.5%.	[55]
9	TiO_2_	50–300 µm	10–20%	FDM	The grain size distribution plays a crucial role in the frequency-dependent variations of the dielectric constant and loss factor in this ceramic composite. These characteristics are essential for its performance in dielectric applications, including its use in capacitors for A/D converters, filtration capacitors, and dielectric resonant antennas.	[56]

The present article reports the trial research results and discussion on possible DLP PUA mechanical properties and chemical durability enhancement with the help of ZnO at maximum applied concentrations. The test results will be useful for successful 3D parts printing made of polymer based-hybrid composites with extended functionality and better mechanical performance.

## 2. Materials and Methods

### 2.1. Applied Materials

The producer is not disclosing the exact composition of the ER Anycubic 3D Printing UV Sensitive Resin, Basic, Clear (Anycubic, Shenzhen, China) for some commercial reasons. The available materials safety data sheet for this product indicates the approximate concentrations of polyurethane acrylate (30–60 wt.%, CAS N° 82116-59-4, epoxy with solvents); isooctyl acrylate (10–40 wt.%, CAS N° 29590-42-9, monomer) and photo initiator (phosphine oxide, 2–5 wt.%). According to the information on producer’s webpage, the liquid form of the ER exhibits density of 1.1 g·cm^−3^, and viscosity of 552 mPa·s. The photo-cured (at the wavelength of 405 ± 8 nm) [57] translucent ER exhibits solid density of 1.284 g·cm^−3^, yield tensile stress up to 23.4 MPa, and elongation up to 14.2% [58].

As-received 99.9 wt.% pure nanometer spherically (episodically) shaped [59] particles containing zinc oxide (ZnO) powder with particle sizes up to 5 µm (Sigma Aldrich, Saint Louis, state of Missouri, USA, product N° 205532, CAS N° 1314-13-2) was used as the filler for production of reinforced photo-cured ER samples [60].

### 2.2. Methods for Samples Manufacturing

The planetary mixer Hauschild Speed Mixer DAC 150.1 FVZ-K (Hamm, Germany) with rotational speed of 1150 rpm was set for 5 min to mix each selected composition with the total weight of 140 g (measured by the laboratory scales KERN EMB-S). The resulting suspension was transported to the printing laboratory and used for DLP within 1 h after mixing. In a series of trials, various DLP attempts were made with different ZnO concentrations to ascertain a dependable printable mixture. Ultimately, a decision was made to produce suspensions with ZnO concentrations of 0.5, 1, 1.5, and 2 wt.%. Additionally, reference samples were printed without additives and subsequently subjected to tensile tests to determine only tensile stress at fracture.

The 3D model for the test specimen was drawn with the help of the AutoCad software (version S.51.0.0 AutoCad 2022) according to the specification for the Type IV described in the standard ASTM D638-14 [61]. The obtained computer aided design (CAD) was virtually sliced with the help of the Photon Workshop V2 1.23 RC8 software, as demonstrated in Table 2.

Settings were chosen for the selected DLP type 3D printer Anycubic Photon Mono (405 nm wavelength, resolution of 1620 × 2560 pixels and pixel size of 51 µm) with detailed specifications demonstrated on the manufacturer webpage [62]. The bottom exposure time of 60 s provided the build plate-resin and initial resin-resin layers adhesion at the beginning of each 3D printing process. The 3D printer was inserted in the insulated box to reduce temperature fluctuation and to stabilize it at +20 °C.

Produced specimens were removed from the build plate, prewashed in isopropanol (99.8%) and immersed in 5 L of isopropanol container and washed with the help of the Anycubic Wash&Cure Machine 2.0 for 10 min. The obtained specimens were additionally cured with UV light (405 nm wavelength, 40 W power) for 10 min inside the same device set in the cure mode. The ZnO containing specimens exhibited the photochromism effect under the applied intensive UV radiation [63].

### 2.3. Methods for Visual and Mechanical Characterization of Materials and Samples

The granulometric composition analysis of the ZnO powder involved the utilization of the laser analyzer Fritsch Analysette 22, manufactured by FRITSCH GmbH in Germany. This analysis was conducted following the dispersion of the powder in isopropanol. Surfaces of produced and tested samples were observed with the help of digital microscope Keyence VHX-2000 (Keyence Ltd., Osaka, Japan) and scanning electron microscope (SEM) TESCAN VEGA (high vacuum mode was selected) and OLYMPUS SZX10 (Olympus Corporation, Tokyo, Japan). Tensile tests of manufactured specimens were performed with the help of tensile testing machine Zwick/Roell Z150 (ZwickRoell GmbH & Co. KG, Ulm, Germany). The applied loading speed was 5 mm·min^−1^. The reference specimens (0 wt.%), fixed in the Zwick/Roell Z020 (ZwickRoell GmbH & Co. KG) tensile testing machine, were subjected to the same loading parameters. The recorded data underwent analysis utilizing the TestXpert software (ZwickRoell GmbH & Co. KG, Ulm, Germany, version II). This software facilitated the determination of the modulus of elasticity by extracting it from the slope of the stress-strain curve within the linear region. The dimensions of specimens were measured with the help of the micrometer screw gauge (±0.005 mm).

### 2.4. Chemical Corrosion Tests

Two different laboratory-scaled chemical corrosion tests were performed to simulate the effect of typical chemically active liquid substances stored in tanker tanks. Produced specimens were immersed in aqueous acetic acid (10%, pH = 5) solution and 1 M NaOH (pH = 12) solution to estimate the specimen’s resistance to acidic and alkaline media, respectively. The universal indicator was used for the measurement of solutions pH. All boxes were sealed, and each corrosion test was left for 7 days at room temperature. Afterward, the samples were extracted from their containers, dried at a constant temperature of 30 °C for one hour until they reached a stable mass, and subsequently subjected to measurement. The loss of mass was measured with the help of the scales KERN EMB-S (KERN & SOHN GmbH, Balingen, Germany) before and after the immersion test.

## 3. Results

### 3.1. Visual and Mechanical Properties of Materials and Samples

#### 3.1.1. ZnO Powder Granulometric Analysis Result

**Figure 1:**
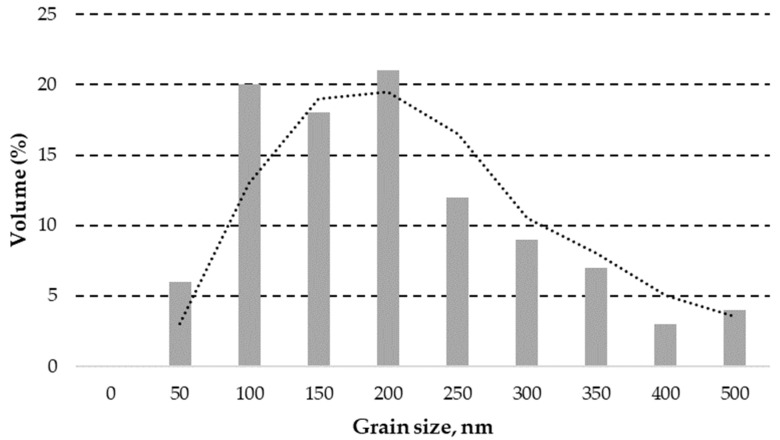
A simplified granulometric analysis of ZnO powder obtained through laser granulometry with two period moving average trendline indicated (dashed line).

The simplified result of granulometric analysis shows that the median particle size (D_50_) in tested ZnO powder is about 177 nm, as demonstrated in Figure 1. The similar particle size distributions (D_50_ = 117 nm) result has been represented by Meng, F.; et.al. for the same ZnO powder product [64].

**Figure 2:**
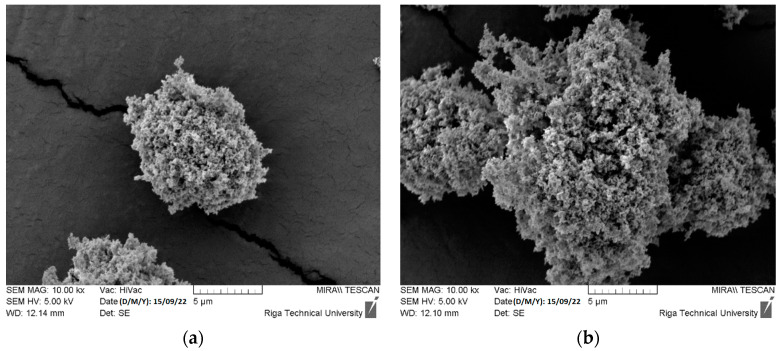
Detected ZnO particles (**a**) and agglomerates (**b**) with particle sizes above 5 µm.

Nevertheless, many particles and their agglomerates with particle sizes above 5000 nm (5 µm) were detected by SEM, as demonstrated in Figure 2a,b. ZnO powder manufacturers recommend employing various deagglomeration methods to achieve target particle sizes for additive manufacturing. These methods, including ultrasonic dispersion, mechanical stirring, or high-shear mixing, can effectively break down agglomerates and ensure the uniform dispersion of particles, ultimately enhancing the quality of the 3D printing feedstock.

#### 3.1.2. Visual Characteristics and Tensile Stress of Manufactured Samples

**Figure 3:**
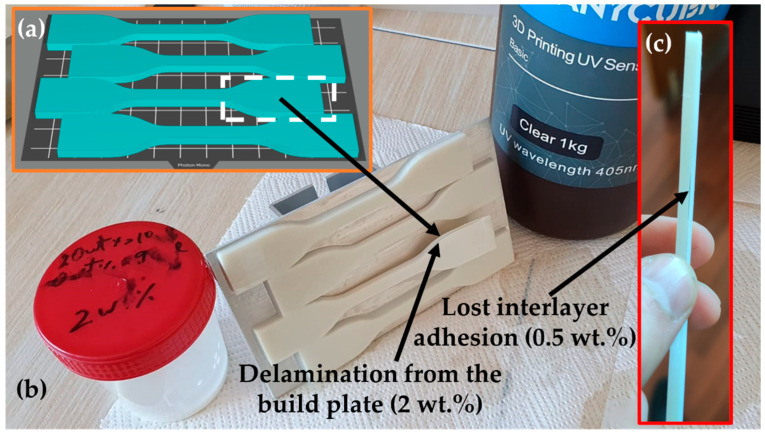
The orientation and layout of CAD objects on the build plate with indicated most failing area (**a**); DLP specimens with defect caused to one of the specimens by the delamination from the build plate (**b**); and interlayer adhesion defect (**c**) caused by transparent film surpassing the adhesion between the build plate and the cured sample.

Some of DLP samples sometimes exhibited various defects such as partial delamination from the same area on the build plate, as shown in the Figure 3a,b. The origin of this event can be attributed to the slightly lower cured ER adhesion force due to the insufficient surface roughness on the working area of the build plate. Hence, in specific regions where the adhesion force between the sample and the transparent film surpasses the adhesion between the build plate and the cured sample, or between cured layers, it can significantly impact the overall quality of the DLP specimen. In rare cases, interlayer adhesion problems were observed during first DLP trials with selected target concentrations. The effect was mitigated by thorough premixing of the suspension before pouring it into the resin vat. Only best quality DLP samples were taken for further testing.

The sufficiently low ZnO aggregation and sedimentation process in the liquid ER provided good quality solid particle distribution in 3D printed objects. However, the largest objects (or thinnest layers) would require longest duration which may lead to significantly heterogenous cured polymer structure. The use of silane agents could potentially improve the maintenance of high Zn nanoparticle dispersion in the DLP polymer matrix [65,66].

The measurements of thickness and width at the center for each specimen are presented in Table 3.

An increase in the concentration of ZnO beyond 0.5 wt.% results in a significant escalation in thickness deviation, ranging from 4.3 times (at 2 wt.%) to a substantial 11.3-fold increase (at 1.5 wt.%). However, the 3D printing of specimens from ER with 1 wt.% ZnO leads to thickness values that are closest to the target values, as compared to other specimens. The average values of all specimens show negative deviation from target thickness value (4 mm). Therefore, possible increase in target thickness (or total height along Z axis) should be considered in CAD design to reach closer desired values.

The mean center width values across all samples exhibit a range from 5.93 mm (0 wt.%) to 6.49 mm (1 wt.%). An increase in ZnO concentration above 0.5 wt.% results in a proportional rise in the average deviation in center width, ranging from 1.5 times (1 wt.%) to 5.5 times (2 wt.%). The average values of all ZnO reinforced specimens show positive deviation from target width at the center value (6 mm). Hence, it is advisable to contemplate a potential reduction in the target center width value or a shorter exposure time during CAD design and DLP printing processes. This approach can bring the final 3D printed object closer to the desired specifications, eliminating the need for additional post-printing material removal.

Fluctuations in sizes and printed parts warping (see Figure 3b) are important errors typically caused by the shrinkage of acrylate-based ERs [67]. Updated recommendations and instructions should be formulated in accordance with research findings and practical experience across various manufacturing conditions. The polymer bond formation between polymerizing material and slightly denser photocured solid polymer and cooling after exothermic chemical reaction caused thermal expansion are two main factors causing the DLP product shrinkage. The remaining internal stress promotes the warping effect in combination with adhesion forces to the transparent film during every retraction process [68]. Reduction in errors and improved mechanical properties can be achieved by careful post processing by heating and photocuring of DLP parts [69].

**Figure 4:**
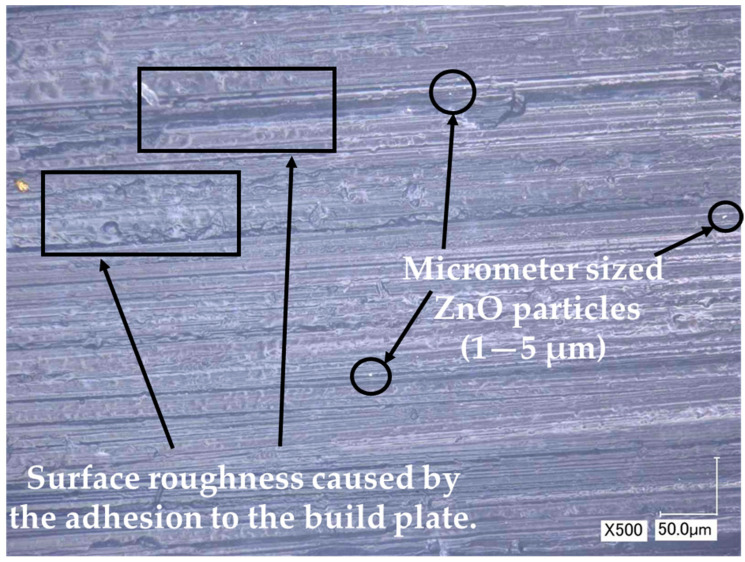
The mimicked rough surface caused from the adhesion to the profiled (textured) build plate and micrometer sized ZnO particles (possible agglomerates) observed by the optical microscope on the surface of 0.5 wt.% ZnO containing sample.

The build plate side of each sample displays the replication of visually observable stripes and defects, as illustrated in Figure 4. These defects are attributed to the presence of several ZnO particles with sizes ranging from 1000 to 5000 nm (from 1 to 5 µm), exhibiting a homogeneous distribution.

**Figure 5:**
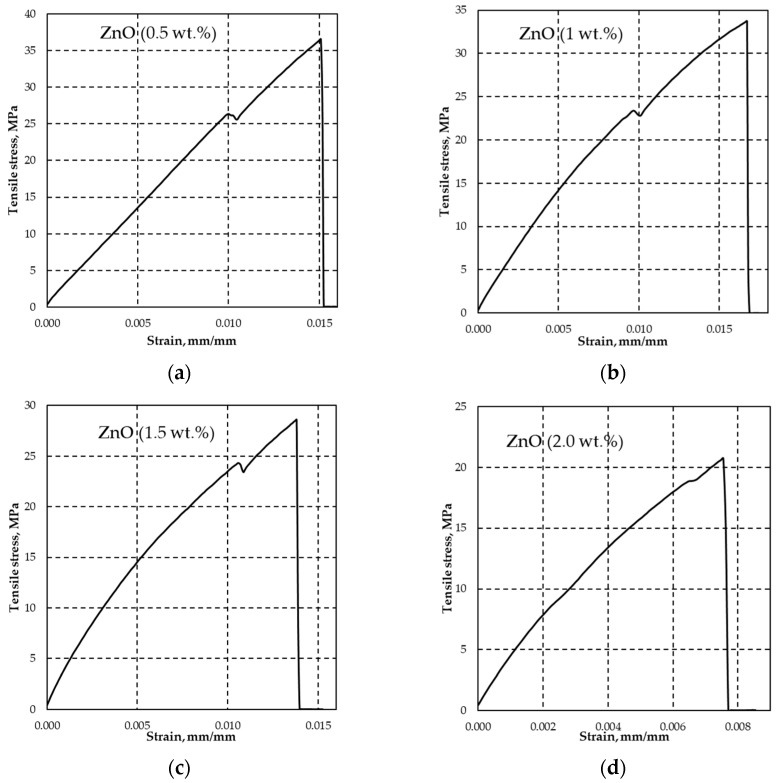
Examples of effect of ZnO concentrations of 0.5 wt.% (**a**); 1 wt.% (**b**); 1.5 wt.% (**c**); and 2 wt.% (**d**) on tensile stress at yield and fracture strains. The increase in ZnO concentrations leads to more brittle composite behavior under applied destructive tensile force.

All DLP composites tested with varying ZnO concentrations exhibit comparable tensile stress at yield, ranging from 23.29 MPa (1 wt.%) to 25.93 MPa (0.5 wt.%), with corresponding elongation/deformation values within the range of 0.0089 to 0.011 mm·mm^−1^, as outlined in Table 4. In contrast, the results of reference samples only provide information on tensile stress at fracture. Notably, it is observed that with higher ZnO concentrations, there is a decrease in tensile stress at fracture. The tensile test measurement curves unequivocally illustrate the increased brittleness of the DLP composite as ZnO concentration is raised, as visually depicted in Figure 5.

**Figure 6:**
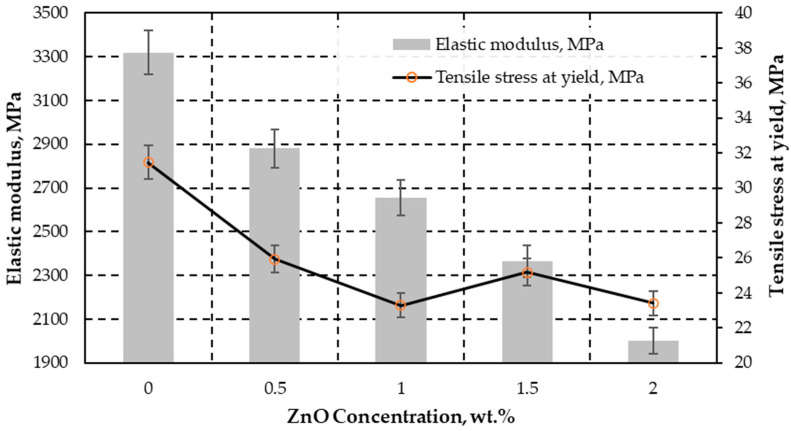
The graph displays the correlation between the elastic modulus (*E*) in megapascals (MPa) on the left *Y*-axis and the tensile stress at the yield point (*σ_y_*) in MPa on the right *Y*-axis. These measurements are analyzed with respect to different ZnO concentrations. Reference sample (0 wt.%) exhibit fracture at yield.

Also, the higher ZnO concentration is the lower the elastic modulus, as shown in Figure 6. This value experiences a reduction of approximately 1.4 times at 0.5 wt.% (reaching 2880 MPa) and 2 wt.% (decreasing to 2001 MPa).

The shape, size, dispersion, and interfacial interaction between ZnO filler and DLP polymer matrix influence the mechanical properties of the product [32,65,66]. The 1 wt.% of tetrapod shaped ZnO whiskers provide similar tensile stress at break (36 MPa) in thin film PUA matrix [70], as compared to present test result (35.4 MPa). The needle shaped ZnO structure would be interesting alternative due to potential DLP matrix reinforcement in case of avoiding the agglomeration and sedimentation without significant impact on surface roughness [27].

#### 3.1.3. DLP ZnO Composites Corrosion Resistance in Acetic Acid Solution (pH = 5)

The ZnO reacts with acetic acid and generates zinc acetate salts according to chemical Equation (1):

2CH_3_COOH + ZnO = Zn(CH_3_COO)_2_ + H_2_O (1)

**Figure 7:**
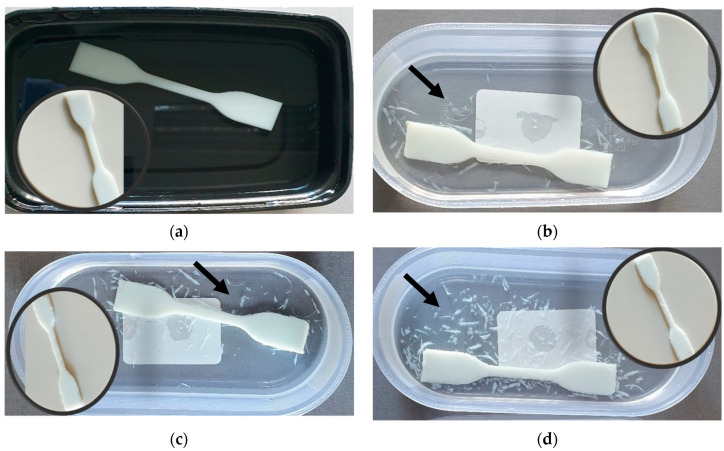
Degradation results after 7 days exposure of polymer composites with ZnO concentrations of 0.5 wt.% (**a**); 1 wt.% (**b**); 1.5 wt.% (**c**); and 2 wt.% (**d**) in acetic acid (pH = 5) solution. Black arrows indicate the degradation products caused by ZnO reaction with acetic acid and destruction of polymer structure by the formation of relatively large crystalline salt (zinc acetate) grains.

**Figure 8:**
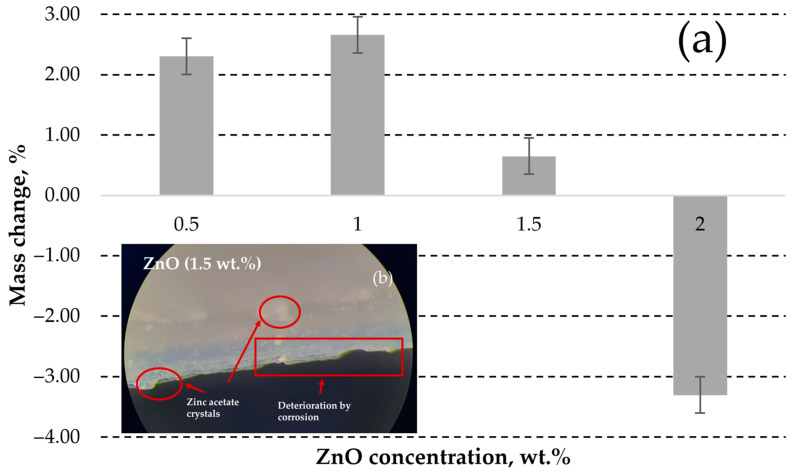
The change of ZnO containing polymer composites mass after exposure in acetic acid for 7 days (**a**); and optical image (magnification ×126) of the corroded 1.5 wt.% sample (**b**) with indicated corrosion products and deterioration defects. Positive values indicate that the gain in mass by corrosion products is higher than the loss of mass by composite deterioration.

**Figure 9:**
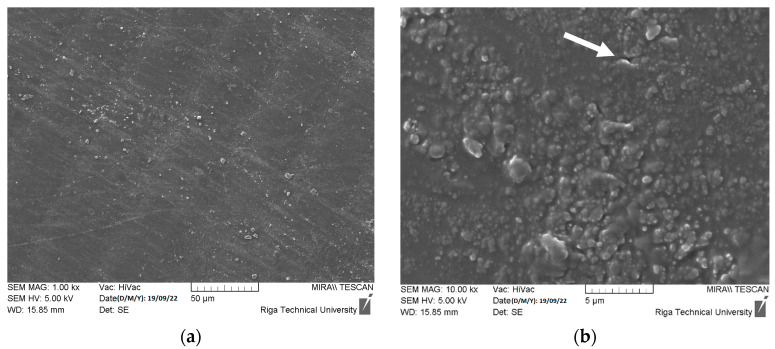
Degradation results after seven days of exposure of polymer composites with ZnO concentration of 1 wt.% in acetic acid (pH = 5) solution captured by SEM with 1000× (**a**) and 10,000× (**b**) resolutions. White arrow indicates the mechanical degradation of cured ER by swelling of ZnO and acetic acid reaction products.

The zinc acetate generated crystal hydrates of Zn(CH_3_COO)_2_(H_2_O)_2_ in the presence of water [71]. The samples with lowest ZnO concentration (0.5 wt.%) exhibited almost no visually observable deterioration signs after immersion in acetic acid, as shown in Figure 7a. The increase in the weight by 2.31% (see Figure 8a) indicates the embedment of almost all generated corrosion products (zinc acetic crystals) in the pores of plastic composites with 0.5 wt.% ZnO. The corrosion of samples with 1, 1.5 and 2 wt.% ZnO leads to detachment of deteriorated plastic particles from the surfaces of exposed materials, as demonstrated in Figure 7b–d, Figure 8b and Figure 9a,b.

The deterioration of 1 and 1.5 wt.% ZnO containing composites also leads to increase in weight of samples (see Figure 8), swelling reaction products increases the surface roughness and cause the formation of new pores on the surface of DLP products, as demonstrated in Figure 9b. Swelling by reaction products occurs when chemical reactions within a material lead to the formation of new compounds with larger molecular structures, causing the material to expand or increase in volume. Therefore, corrosive solution can access more ZnO particles under the corroded surface. This effect indicates the exposure of higher ZnO particles to reactive environment and embedment in the corroded surface pores. Despite the solubility of 43 g·100 mL_water_^−1^ [72], the zinc acetate preservation occurred due to specimen immersion in the standing aqueous solution. Therefore, corrosion products significantly limited the penetration of the corrosive substance to fresh ZnO sites inside the DLP composites. Additionally, the free water can remain mechanically trapped in the porous structure of the deteriorated DLP composites after removing the samples from aqueous solutions and drying.

However, a significant weight loss was observed in the case of the sample containing 2 wt.% ZnO, attributed to the intensive detachment of deteriorated material, as illustrated in Figure 7d.

#### 3.1.4. DLP ZnO Composites Corrosion Resistance in Sodium Hydroxide Solution (pH = 12)

The ZnO reacts with aqueous sodium hydroxide solution and generates sodium tetrahydroxozincate according to chemical Equation (2):

2NaOH + ZnO + H_2_O = Na_2_[Zn(OH)_4_] (2)

**Figure 10:**
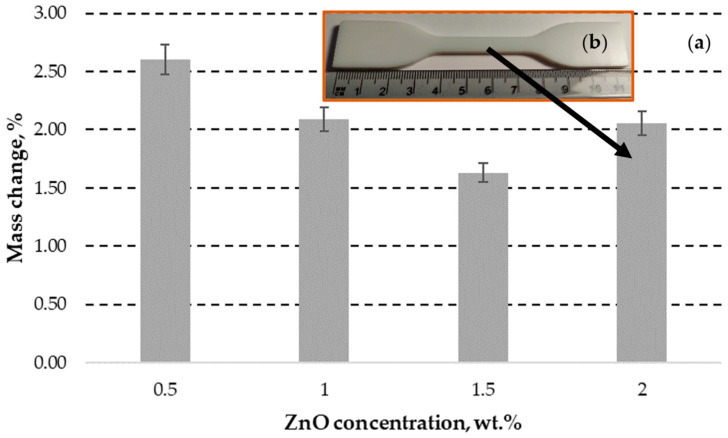
The increase in ZnO containing polymer composites mass after exposure in the aqueous 1 M NaOH solution (pH = 11) for 7 days (**a**); and photo of the corroded 2 wt.% ZnO containing sample without significant impact on surface morphology (**b**).

The sodium tetrahydroxozincate also forms crystal hydrates (Na_2_[Zn(OH)_4_]·2H_2_O) in the presence of aqueous solution. The corrosion products and mechanically trapped water inside the defects of DLP printed ZnO composites leads to increase in weight of corroded samples by up to 2.6%, as demonstrated in Figure 10a.

**Figure 11:**
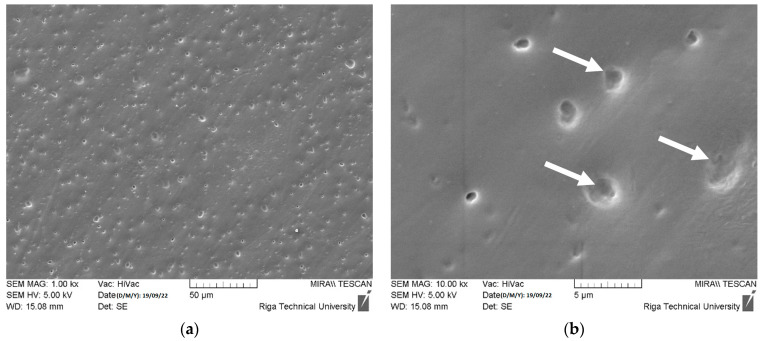
Degradation results after 7 days exposure of polymer composites with ZnO concentration of 2 wt.% in 1 M NaOH solution (pH = 11) solution captured by SEM with 1000× (**a**) and 10,000× (**b**) resolutions. The reaction leads to the formation of smooth particles (indicated by arrows) with passivation effect.

Remarkably, that increase in ZnO concentration up to 1 and 1.5 wt.% leads to slightly lower increase in mass change, as compared to 0.5 wt.% specimen. This effect can be attributed to the more efficient block of defects by corrosion products in standing (stagnated) aqueous NaOH solution, as observed by SEM (see Figure 11a,b). Reaction leads to formation of smoother grains as compared to fresh ZnO powder. However, the specimen with 2 wt.% ZnO exhibits similar mass change (2.05%) as compared to the specimen with 1 wt.% ZnO (2.09%). This effect indicates the limit of blocking effect by ZnO at concentrations between 1.5 and 2 wt.% in tested conditions. However, the NaOH and reaction products lead almost no effect on visually observable shape and morphology of any sample, as demonstrated in Figure 10b.

The ester groups of acrylic polymer and acrylic styrene copolymer hydrolyses into carboxylate salt of sodium [73]. However, in case of tested PUA composites, the surface of polymer material passivates the chemical corrosion. Further detailed studies are necessary to provide a better understanding of corrosion mechanisms.

## 4. Discussion

The DLP method gets attention due to its operational simplicity and for quickly obtainable outputs. Unfortunately, limited supplementing (raw) materials availability on the market [74] and manufacturing precision (spatially controlled solidification) [75] leads to demand for intensive R&D process to obtain instructions for profitable and reliable materials and methods. These factors contribute to the production speed, product durability, and the miniaturization of objects for DLP printing, particularly for small-scale and highly detailed items [76].

The observed dimensional variations (thickness and width, see Table 3) in a 3D-printed structure using DLP printing with ZnO reinforcement in PUA are influenced by multiple factors affecting the final product’s dimensions. The polymerization and crosslinking of PUA may cause *Z*-axis shrinkage, while the addition of ZnO nanoparticles can induce expansion along the X and Y axes [77]. Uneven curing, post-curing conditions, ZnO distribution, and layer orientation contribute to differential dimensional changes. Mitigating photocuring shrinkage is essential for enhancing material performance. Various methods, including hyperbranched polymers and thiol-ene photopolymerization, have proven effective [78]. All of these aspects should be researched in detail in further studies.

Obtained DLP PUA with ZnO additives provide stable and durable protective solutions for both indoor [79] and outdoor [80] conditions. However, the chemical durability under different corrosive conditions typical for chemical transportation and storage tanks, household chemical containers, and many other exposed compartments should be studied in detail. The demonstrated strong corrosion resistance when subjected to a tested sodium hydroxide solution with a pH of 12 not only expands the potential applications of this composite to include the transportation of alkaline solutions and dry substances but also paves the way for its utilization in groundbreaking construction materials characterized by a high pH. One notable example is the development of innovative building materials, such as a 3D printed concrete composite based on a Portland cement binder [81].

An innovative approach involves hybrid concrete-DLP 3D printers, allowing for the incorporation of ER and polymer elements (e.g., polymer films and fibers) [82,83] during the concrete deposition process. These printers could come in various sizes to accommodate polymer reinforced lightweight concrete based [84] large-scale cylindrical tanks [85] and other construction. ZnO reinforcement in ERs can sustain alkaline corrosion during concrete hardening during curing process and periodical wetting during service period. Thus, both concrete properties can be improved, and new functions can be provided.

**Figure 12:**
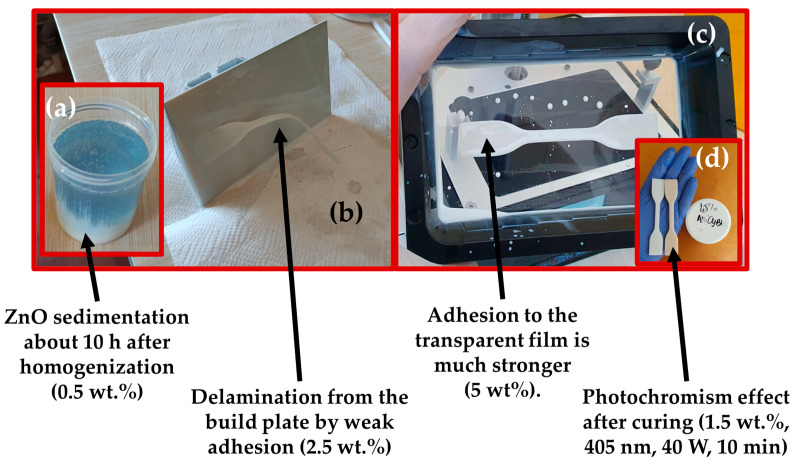
The sedimentation (white colored substance) of the ZnO in raw ER (**a**); first layer delamination from the build plate (**b**); DLP failure by too strong first layer adhesion to the transparent film (**c**); and photochromism effect after high power (40 W) UV curing (**d**).

The sedimentation of the ZnO powder become observable inside the container about 10 h after mixing with the planetary mixer, as demonstrated in Figure 12a. The maximum ZnO concentration for the successful 3D printing was preliminarily detected by studying the range of concentrations from 0.5 to 10 wt.% and it was found that materials with content of 2 wt.% or lower are enabling to produce multilayered structure without delamination from the build plate and stronger adhesion to the transparent film, as demonstrated in Figure 12b,c.

Successfully achieving product printing with a relatively low concentration of ZnO powder (from 0.5 to 2 wt.%) highlights the imperative for thorough multifactorial studies. Subsequent investigations are poised to elevate concentrations of ZnO and other fillers up to 52 wt.%, as depicted in Table 1. This approach not only promises enhanced properties for the DLP product but also opens avenues for novel functional attributes.

The attachment of active ER premixing system would reduce the sedimentation effect on 3D printing performance. Further studies need to test different active mixing techniques.

When ZnO-reinforced polymers exhibit the photochromism effect, it can significantly enhance their aesthetic properties. Photochromic materials, such as those containing ZnO nanoparticles, can change color or optical characteristics in response to UV light, as demonstrated in Figure 12d. For example, this effect can be valuable for shielding polymers from excessive UV exposure during the disinfection process of tanker walls.

In-depth studies on the behavior of ZnO-reinforced PUA composites under shock loading conditions (e.g., pendulum impact test) are also required in future studies. Stukhlyak et al. conducted such research exploring the impact of meso- and macroscale processes on the dynamic fracture mechanisms of cross-linked epoxy composites [28]. This involves cross-linking during the curing of thermosetting plastics, transforming epoxy resins into cross-linked polymers through the polymerization of polyfunctional monomers or oligomers. The cross-bonding of linear and branched macromolecules with reactive groups of an epoxy polymer is a crucial aspect, with cross-links in these polymers being chemical, physical, and topological [86].

Predicting PUA based composite behavior involves accounting for microstructural complexity. In nanocomposites, non-dimensional models show no significant nanoscopic size effect below the transition temperature, but above it, a notable discrepancy emerges between experimental modulus and theory. This suggests negligible effects in the glassy state become relevant in the rubbery domain, necessitating evaluation of mechanical interactions using multi-scale approaches or finite element modeling. This aligns with growing industrial interest for potential applications in sporting equipment, automotive parts, and packaging due to high barrier properties and enhanced flame retardance. Industrial nanocomposites, mainly using rubbery, thermosets, or semi-crystalline matrices, exhibit typical viscoelastic behaviors [87].

When using ZnO powder as a filler in DLP photocured resins, it’s essential to consider its ecological impact, including proper disposal and recycling measures to mitigate potential environmental consequences.

## 5. Conclusions

As-received 99.9 wt.% pure ZnO powder with D_50_ = 177 nm and maximum particle (including agglomerate) sizes up to about 5 µm were mixed in commercially available polyurethane acrylate, isooctyl acrylate, and phosphine oxide mixture with ZnO concentrations from 0.5 up to 2 wt.% (from 0.5 to 10 wt.%, during preliminary studies). Adhesion weakening to the build plate and next layer (stronger adhesion to transparent film) limits the possibility to increase the ZnO concentration under selected DLP settings. The change in ZnO concentration causes thickness deviations in DLP samples. The 1 wt.% samples achieve closest value of thickness (3.99 ± 0.16 mm) to target thickness value (4 mm), while 0.5 wt.% ZnO containing samples exhibit lowest deviation in average thickness (±0.03 mm).

Nonetheless, the deviation in the width of the DLP product consistently escalates, increasing by a factor of 1.5–5.5 times as the concentration rises from 1 wt.% to 2 wt.%. This results in an exceeding of the target value of 6 mm in the horizontal plane for all concentrations, with a minimum overage of 0.33 ± 0.02 mm. To address this deviation, it can usually be corrected by simply reducing the exposure time while keeping the initially set conditional parameters intact.

All tested DLP composites with ZnO exhibits similar tensile stress from 23.29 (1 wt.%) up to 25.93 MPa (0.5 wt.%) at similar elongation/deformation values (0.0089 up to 0.0110), however, the increase in ZnO concentration increase brittleness of the product (tensile stress at fracture reduces to 24.04 MPa and elastic modulus reduces to 2001 MPa at ZnO concentration of 2 wt.%).

The increase in ZnO concentration increases the total area for reaction with acetic acid solution and causes visually observable loss of ER material from DLP products from 1 wt.% ZnO. The reaction products (zinc acetate salts) swell and cause formation of new defects under the surface layer. Therefore, use of ZnO reinforced DLP resin is limited to acidic environments.

All samples exhibit good corrosion resistance to applied aqueous sodium hydroxide solution (pH = 12). The reaction products (mainly generates sodium tetrahydroxozincate) form on the surface of specimens and passivates further reaction when subjected to standing (stagnating) solution. Reaction products cause smooth surface above the ZnO, therefore reduces the possible friction caused defects. Future studies should include the measurement and reporting of roughness values.

Generally, the produced samples are suitable for application in alkaline environment and can be applied as protective coatings over tank walls or used as structural or functional elements (e.g., in Portland cement concrete walls).

## Figures and Tables

**Table 2:** Slice settings for manufacturing specimens with the help of the Anycubic Photon Mono 3D printer. Printing time of one sample was approximately 15 min (demonstrated by DLP printer).

Slice Setting Parameter	Value	Unit of Measure
Layer thickness	50	µm
Normal exposure time	2	s
Off time	0.5	s
Bottom exposure time	40	s
Bottom layers	6	layers
Z axis lift distance (after printing of each layer)	6	mm
Z axis lift speed	5	mm·s^−1^
Z axis retract speed	6	mm·s^−1^

**Table 3:** The effect of ZnO concentrations on average thicknesses and widths at the centers of 3D printed specimens and average deviation from target thickness (4 mm) and target width (6 mm) values. A minimum of five samples were measured for each material.

	ZnO Concentration				
	0 wt.%	0.5 wt.%	1 wt.%	1.5 wt.%	2 wt.%
Average thickness, mm (deviation, mm)	4.01 (±0.07)	3.84 (±0.03)	3.99 (±0.16)	3.72 (±0.34)	3.83 (±0.12)
Average deviation from the target thickness, mm	(~0.000)	−0.160	−0.003	−0.280	−0.170
Average width at the center, mm (deviation, mm)	5.93 (±0.03)	6.33 (±0.02)	6.49 (±0.03)	6.42 (±0.07)	6.47 (±0.11)
Average deviation from the target width at the center, mm	−0.070	+0.330	+0.490	+0.420	+0.470

**Table 4:** The average tensile stress at yield and fracture loads of ZnO containing DLP composites. A minimum of five samples were measured for each material.

	Sample				
	0 wt.%	0.5 wt.%	1 wt.%	1.5 wt.%	2 wt.%
Tensile stress at yield, *σ_y_* (MPa)	-	25.93 (±0.44)	23.29 (±0.18)	25.19 (±1.25)	23.41 (±3.21)
Elongation/deformation at yield, *ε* (mm·mm^−1^)	-	0.0091 (±0.0014)	0.0089 (±0.0012)	0.0110 (±0.0001)	0.0091 (±0.0036)
Tensile stress at fracture, *σ_UTS_* (MPa)	43.1 (±4.06)	38.76 (±0.03)	35.40 (±0.03)	29.93 (±0.03)	24.04 (±0.03)
Elongation/deformation at fracture, *ε_UTS_* (mm·mm^−1^)	-	0.0156 (±0.0001)	0.0172 (±0.0007)	0.0146 (±0.0011)	0.0112 (±0.0051)

## Data Availability

Data are contained within the article.
